# Supplementary figures and images for: Genomic analysis of variability in Delta-toxin levels between Staphylococcus aureus strains
Source: PeerJ. 2020 Mar 24;8:e8717. doi: 10.7717/peerj.8717 (PMC7100594; doi:10.7717/peerj.8717)

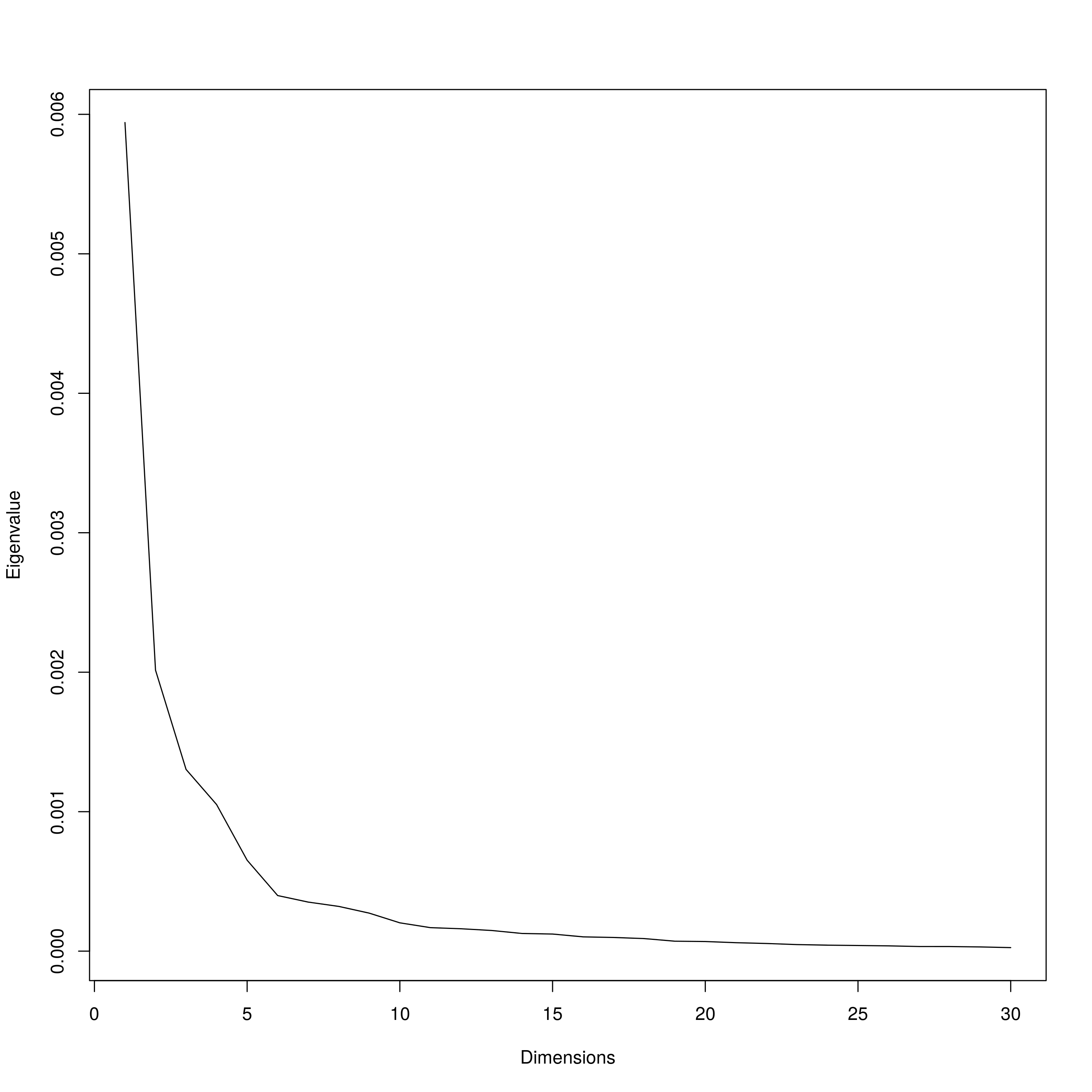

Supplement: Figure S1 — For SEER, a distance matrix was estimated from kmers from S. aureus genome assemblies. To determine the number of dimensions to project the distance matrix into to estimate population structure, eigenvalues were plotted against dimensions. Six dimensions was chosen based on the second ”elbow” of the graph. [file peerj-08-8717-s001.png]

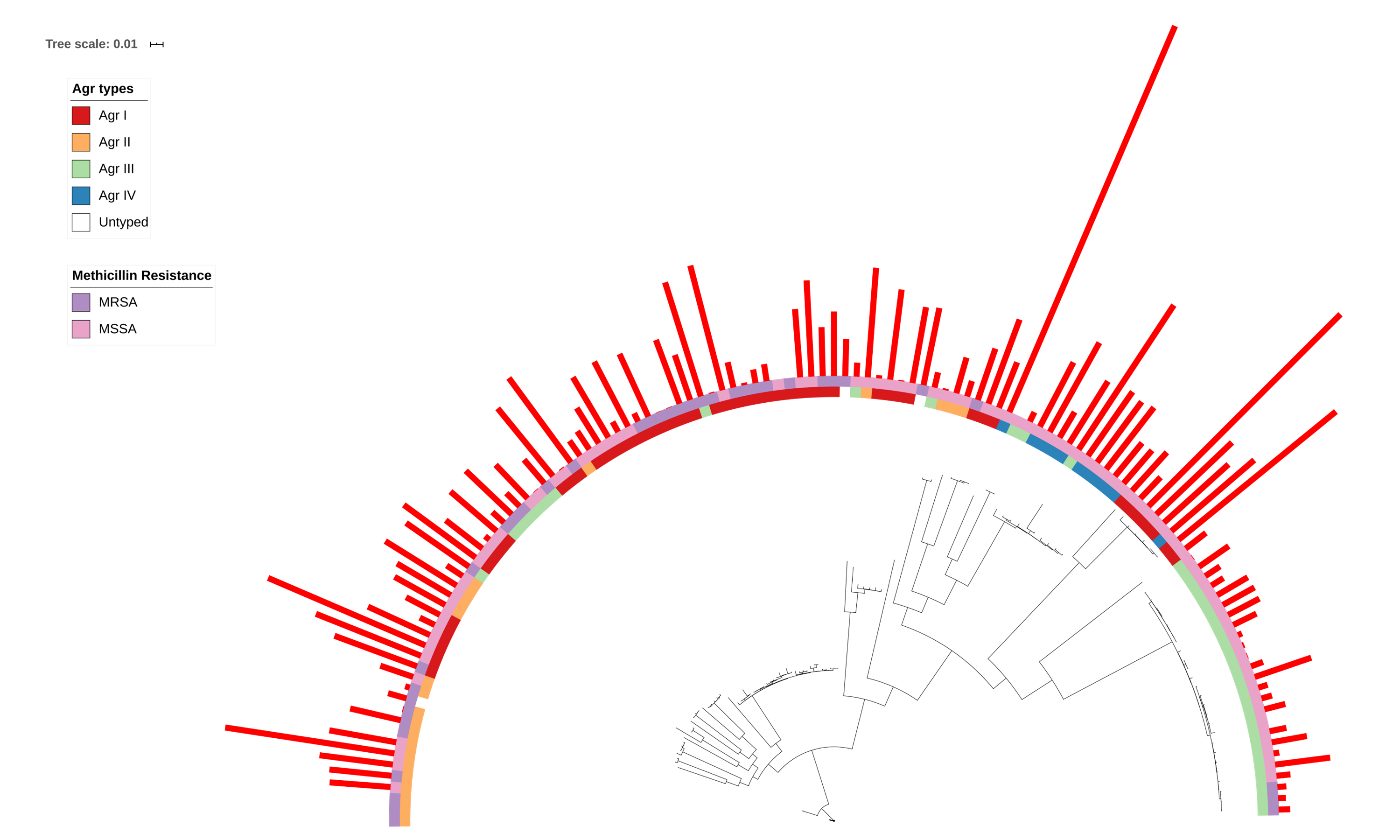

Supplement: Figure S2 — 124 S. aureus strains in 23 clonal complexes (CC) were used to create a core genome phylogeny using RAxML. Agr type is represented in the inner ring and color coded. MRSA/MSSA status is represented in the outer ring and color coded. Red bars represent δ-toxin values from HPLC. Missing bars indicate δ-toxin was undetectable by HPLC. [file peerj-08-8717-s002.png]

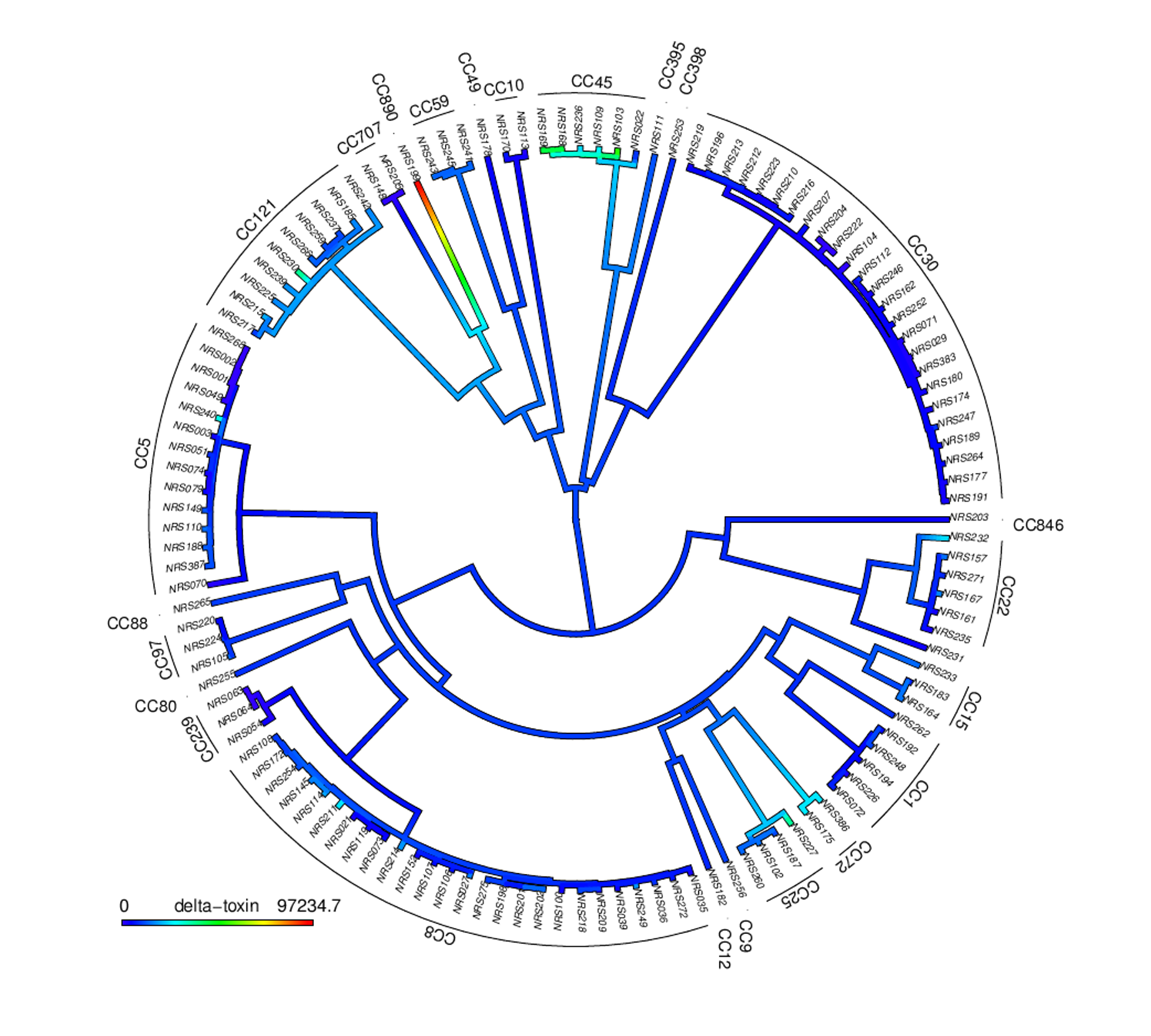

Supplement: Figure S3 — The julia package PhyloNetworks was used to reconstruct ancestral states of δ-toxin based on 124 S. aureus strains. [file peerj-08-8717-s003.png]
