# Supplementary material for: Genomic analysis of variability in Delta-toxin levels between Staphylococcus aureus strains
Source: PeerJ. 2020 Mar 24;8:e8717. doi: 10.7717/peerj.8717 (PMC7100594; doi:10.7717/peerj.8717)
Supplement: Table S2 [file peerj-08-8717-s005.docx]

| **Primer** | **Sequence (5’ -> 3’)** | **Description** |
| --- | --- | --- |
| Tn_Up | CTCGATTCTATTAACAAGGG | Designed by authors of NTML. For use if transposon is in plus orientation with gene specific primer. 464 bp to transposon end. |
| Tn_Buster | GCTTTTTCTAAATGTTTTTTAAGTAAATCAAGTAC | Designed by authors of NTML. For use if transposon is in minus orientation with gene specific primer to transposon end. |
| fadD_tn | ATCAGAGAAGAAACGTGC | Gene specific primer for *fadD* (SAUSA300_0227). Used to check transposon insertion in NE260. |
| agrA_tn | TTTTTAACGTTTCTCACCGAT | Gene specific primer for *agrA* (SAUSA300_1992). Used to check transposon insertion in NE1532. |
| brnQ_tn | TTAAGTTGTCGCTTGTTTCG | Gene specific primer for *brnQ* (SAUSA300_0306). Used to check transposon insertion in NE605. |
| carA_tn | TTAGGCATTGATATGACGC | Gene specific primer for *carA* (SAUSA300_1095). Used to check transposon insertion in NE1526. |
| glpD_tn | TTTTGCTCTACAAACGCAT | Gene specific primer for *glpD* (SAUSA300_1193). Used to check transposon insertion in NE233. |
| hemL_tn | TACGACTTAAAGCCGTATC | Gene specific primer for *hemL* (SAUSA300_1614). Used to check transposon insertion in NE303. |
| hlgB_tn | TCACTTTGTGATTTTCCCAA | Gene specific primer for *hlgB* (SAUSA300_2367). Used to check transposon insertion in NE1682. |
| isdC_tn | TTATTCCACATTGCCTTTAGAT | Gene specific primer for *isdC* (SAUSA300_1030). Used to check transposon insertion in NE557. |
| NE151_tn | TTATCCTGCATTCTTTGACTC | Gene specific primer for hi77 ORF109-like protein (SAUSA300_1928). Used to check transposon insertion in NE151. |
| sbnC_tn | GCATTTTTGTACACGTCC | Gene specific primer for *sbnC* (SAUSA300_0120). Used to check transposon insertion in NE1031. |
| thiD_tn | AATTCATCGTCTAATCCCTCT | Gene specific primer for *thiD* (SAUSA300_2049). Used to check transposon insertion in NE1428. |
| carA_pOS1_rev | CACTAGATAACGTTTGCTTTGCAT-GCATATGTTCACCTCAATTGTATTTATCCC | SOE primer to amplify linearized pOS1-Plgt with *carA* overhangs |
| carA_pOS1_fwd | GGAGCGTCATATCAATGCCTAA-TCGAGGATCCAAACAAGGGGG | SOE primer to amplify linearized pOS1-Plgt with *carA* overhangs |
| pOS1_carA_fwd | GGGATAAATACAATTGAGGTGAACATATGC-ATGCAAAGCAAACGTTATCTAGTG | SOE primer to amplify *carA* with pOS1-Plgt overhangs |
| pOS1_carA_rev | CCCCCTTGTTTGGATCCTCGA-TTAGGCATTGATATGACGCTCC | SOE primer to amplify *carA* with pOS1-Plgt overhangs |
