# Supplementary material for: Genomic analysis of variability in Delta-toxin levels between Staphylococcus aureus strains
Source: PeerJ. 2020 Mar 24;8:e8717. doi: 10.7717/peerj.8717 (PMC7100594; doi:10.7717/peerj.8717)
Supplement: Table S3 — Softwares used were SEER, bugwas, treeWAS, and DBGWAS. S. aureus N315 was used as reference genome to map GWAS hits. [file peerj-08-8717-s006.docx]

| **Sequence** | **Amino acid** | **N315 mapping** | **Protein/Gene** | **GWAS method** |
| --- | --- | --- | --- | --- |
| CAAATACAATACCAATGACACGTCGATTGCTAATGACTATTTTAATAAACCGGCAAAGTACATTAAGAAAAATGGTAAATTGTATGTTCAAATAACTGTAAACCACAGTCATTGGATTACAGGAATGAGTAT  A -> G  C -> A  T -> A | S47S  V72V  T79T | 1108175 - 1108306  (33 kmers)  1108199  1108274  1108295 | [WP_000789821.1](https://www.ncbi.nlm.nih.gov/protein/446712477)  isdC | SEER  Binary phenotype |
| CCAGCTCAAATTGAGGCTTATACTGAAGAAGTTGAGCAAGCAATTAAAGAAGCGCAACATGGAAATAATCAACCAGCAGTTAAAGAATAATTAATTTGTACAATCATAAACTG  A -> G  A -> T | E533E  A534A | 1300876 - 1300988  (14 kmers)  1300890  1300893 | [WP_001218596.1](https://www.ncbi.nlm.nih.gov/protein/447141340)  glpD | SEER  Binary phenotype |
| ACAGTCTTTTCAGTTGATTCTGAAATACCATCTAAATCTACTTCTGCATCAACTTCGCTTATATTTTGCAAGTCATCTCGACTACCGAAGATTTCAATTTCTTTATCTTCTAAGTCAATCGAACTTAATTCTTTATCATCTGCTAAACTACCTTTCTGTTTAACATTTACTTTAACCTTTTTGCTA  T -> C  A -> G  C -> T | L264L  D249D  E244E | 2227323 - 2227508  (87 kmers)  2227391  2227436  2227451 | [WP_000894032.1](https://www.ncbi.nlm.nih.gov/protein/446816776)  YbbR-like domain-containing protein | SEER  Binary phenotype |
| C -> T | N220N | 1182697 | [WP_001190913.1](https://www.ncbi.nlm.nih.gov/protein/447113657)  carA | Bugwas  Binary phenotype |
| T -> C (complement) | D194D | 349263 | [WP_000745087.1](https://www.ncbi.nlm.nih.gov/protein/446667741)  brnQ | Bugwas  Binary phenotype |
| T -> C  C -> T  G -> A | D426D  P643L  A663A | 473701  474351  474412 | [WP_000211545.1](https://www.ncbi.nlm.nih.gov/protein/446133690)  DUF2309 family protein | Bugwas  Binary phenotype |
| T -> C | L242L | 2481094 | [WP_000783428.1](https://www.ncbi.nlm.nih.gov/protein/446706082)  hlgB | Bugwas  Binary phenotype |
| G -> A (complement)  A -> G (complement) | M435I  K436E | 2380169  2380168 | [WP_000451828.1](https://www.ncbi.nlm.nih.gov/protein/446373973)  Na+/H+ antiporter NhaC family protein | Bugwas  Binary phenotype |
| T -> A (complement) | D83E | 2532805 | [WP_001229087.1](https://www.ncbi.nlm.nih.gov/protein/447151831)  opp1A | Bugwas  Binary phenotype |
| T -> A | R282R | 26493 | [WP_000871607.1](https://www.ncbi.nlm.nih.gov/protein/446794351)  walK | Bugwas  Binary phenotype |
| A -> T (complement) | P4P | 1037680 | [WP_000104254.1](https://www.ncbi.nlm.nih.gov/protein/446026399)  qoxD | Bugwas  Binary phenotype |
| A -> G | V84V | 299201 | [WP_000610204.1](https://www.ncbi.nlm.nih.gov/protein/446532858)  tarJ | Bugwas  Binary phenotype |
| C -> T | V467V | 132180 | [WP_001179385.1](https://www.ncbi.nlm.nih.gov/protein/447102129)  sbnC | Bugwas  Binary phenotype |
| T -> C (complement)  G -> T (complement) | R259R  T321T | 1706627  1706441 | [WP_000472302.1](https://www.ncbi.nlm.nih.gov/protein/446394447)  clpX | Bugwas  Binary phenotype |
| A -> G | D146G | 546992 | [WP_000613722.1](https://www.ncbi.nlm.nih.gov/protein/446536376)  folK | Bugwas  Binary phenotype |
| C -> T (complement)  T -> C (complement)  G -> A (complement) | G98G  V116V  T164T | 1710281  1710227  1710083 | [WP_000032657.1](https://www.ncbi.nlm.nih.gov/protein/445954802)  NUDIX domain-containing protein | Bugwas  Binary phenotype |
| G -> A (complement)  C -> T (complement)  C -> T (complement)  C -> A,T (complement) | V227V  I251I  T265T  G285G | 1702275  1702203  1702161  1702101 | [WP_001230232.1](https://www.ncbi.nlm.nih.gov/protein/WP_001230232.1)  hemC | Bugwas  Binary phenotype |
| G -> A (complement) | T299T | 1699418 | [WP_001270865.1](https://www.ncbi.nlm.nih.gov/protein/WP_001270865.1)  hemL1 | Bugwas  Binary phenotype |
| T -> C (complement) | N263N | 272940 | [WP_001008400.1](https://www.ncbi.nlm.nih.gov/protein/446931144)  fadE | Bugwas  Binary phenotype |
| G -> A (complement) | A102A | 1680683 | [WP_000457386.1](https://www.ncbi.nlm.nih.gov/protein/446379531)  50S ribosomal protein L21 | Bugwas  Binary phenotype |
| A -> G (complement) | G277G | 1676466 | [WP_001005768.1](https://www.ncbi.nlm.nih.gov/protein/446928512)  ruvB | Bugwas  Binary phenotype |
| T -> A  T -> C | S146S  C179C | 1697413  1697512 | [WP_001001237.1](https://www.ncbi.nlm.nih.gov/protein/446923981)  tag | Bugwas  Binary phenotype |
| C -> T, A (complement) | R353R | 271053 | [WP_000142198.1](https://www.ncbi.nlm.nih.gov/protein/446064343)  fadD | treeWAS |
| A -> G | K116K | 2803540 | [WP_000154162.1](https://www.ncbi.nlm.nih.gov/protein/446076307)  vraD | treeWAS |
| A -> G (complement) | L69L | 316218 | [WP_000757880.1](https://www.ncbi.nlm.nih.gov/protein/446680534)  degA | treeWAS |
| T -> C | V549V | 19974 | [WP_001081640.1](https://www.ncbi.nlm.nih.gov/protein/447004384)  cyclic-di-AMP phosphodiesterase | treeWAS |
| G -> A | G520G | 240930 | [WP_000236427.1](https://www.ncbi.nlm.nih.gov/protein/446158572)  ggt | treeWAS |
| A -> G, T (complement)  C -> T (complement) | V287V  Y319Y | 2527215  2527119 | [WP_000675401.1](https://www.ncbi.nlm.nih.gov/protein/446598055)  MFS transporter | treeWAS |
| T -> G | P347P | 1306337 | [WP_000652047.1](https://www.ncbi.nlm.nih.gov/protein/446574701)  aminotransferase class I/II-fold pyridoxal              phosphate-dependent enzyme | treeWAS |
| T -> A | V360V | 885317 | [WP_001074405.1](https://www.ncbi.nlm.nih.gov/protein/446997149)  sufB | treeWAS |
| C -> T | D113D | 2698328 | [WP_000198598.1](https://www.ncbi.nlm.nih.gov/protein/446120743) | treeWAS |
| C -> A,T (complement) | H181N, H181Y | 312161 | [WP_000031262.1](https://www.ncbi.nlm.nih.gov/protein/445953407)  class I SAM-dependent methyltransferase | treeWAS |
| G -> T, A (complement) | G196V, G196D | 2148703 | [WP_000594954.1](https://www.ncbi.nlm.nih.gov/protein/446517608)  thiD | treeWAS |
| GATAGGGGCTATTTTAATAAAATTCGTCCTC | N/A | 1383811 - 1383841 | Intergenic, 18 bp into end of [WP_000523668.1](https://www.ncbi.nlm.nih.gov/protein/446445813)  SWIM zinc finger family protein | DBGWAS |
